# Examining organisational responses to performance-based financial incentive systems: a case study using NHS staff influenza vaccination rates from 2012/2013 to 2019/2020

**DOI:** 10.1136/bmjqs-2021-013671

**Published:** 2021-09-28

**Authors:** Adiba Liaqat, Suzy Gallier, Katharine Reeves, Hannah Crothers, Felicity Evison, Kelly Schmidtke, Paul Bird, Samuel I Watson, Kamlesh Khunti, Richard Lilford

**Affiliations:** 1 Health Informatics, University Hospitals Birmingham NHS Foundation Trust, Birmingham, UK; 2 Warwick Medical School, University of Warwick, Coventry, Coventry, UK; 3 Institute for Translational Medicine, University Hospitals Birmingham NHS Foundation Trust, Birmingham, UK; 4 West Midlands Academic Health Science Network, Birmingham, UK; 5 Institute of Applied Health Research, University of Birmingham, Birmingham, UK; 6 Diabetes Research Centre, University of Leicester, Leicester, UK

**Keywords:** financial incentives, performance measures, healthcare quality improvement

## Abstract

**Objective:**

Financial incentives are often applied to motivate desirable performance across organisations in healthcare systems. In the 2016/2017 financial year, the National Health Service (NHS) in England set a national performance-based incentive to increase uptake of the influenza vaccination among frontline staff. Since then, the threshold levels needed for hospital trusts to achieve the incentive (ie, the targets) have ranged from 70% to 80%. The present study examines the impact of this financial incentive across eight vaccination seasons.

**Design:**

A retrospective observational study examining routinely recorded rates of influenza vaccination among staff in all acute NHS hospital trusts across eight vaccination seasons (2012/2013–2019/2020). The number of trusts included varied per year, from 127 to 137, due to organisational changes. McCrary’s density test is conducted to determine if the number of hospital trusts narrowly achieving the target by the end of each season is higher than would be expected in the absence of any responsiveness to the target. We refer to this bunching above the target threshold as a ‘threshold effect’.

**Results:**

In the years before a national incentive was set, 9%–31% of NHS Trusts reported achieving the target, compared with 43%–74% in the 4 years after. Threshold effects did not emerge before the national incentive for payment was set; however, since then, threshold effects have appeared every year. Some trusts report narrowly achieving the target each year, both as the target rises and falls. Threshold effects were not apparent at targets for partial payments.

**Conclusions:**

We provide compelling evidence that performance-based financial incentives produced threshold effects. Policymakers who set such incentives are encouraged to track threshold effects since they contain information on how organisations are responding to an incentive, what enquiries they may wish to make, how the incentive may be improved and what unintended effects it may be having.

## Introduction

Performance-based financial incentive systems are widely used to improve performance levels in healthcare organisations.^1 2^ Examples of healthcare systems that use performance-based financial incentives include Medicare’s Quality Payment Program in the USA^3^ and the National Health Service’s (NHS) Commissioning for Quality and Innovation (CQUIN) programme in England.^1^ The literature on the effectiveness of financial incentives, and the many ways they may backfire, is extensive.^4^

Performance-based incentive systems often rely on the data that partner organisations collect, process and report about their outcomes (eg, readmission rates) or processes (eg, medication review rates). Either way, the incentive system must establish a target performance level that will trigger the incentive along with a census date by which that target must be reached. Organisations may respond to the target irrespective of how close they are to reaching it or they may titrate their response. Psychological theories anticipate the latter, as organisations on a trajectory to fall just short of their target may take special steps to remedy the situation.^5^ If these steps succeed, then a discontinuous pattern may appear in the distribution of reported performance levels as a sharp increase in the density function of performance at the target value.^6^ In the present paper, we refer to this discontinuous pattern or ‘bunching’ as ‘threshold effects’.

Two types of organisational behaviour may underlie threshold effects. First, some organisations may apply effort to genuinely achieve a target while others are discouraged from making further efforts, either because they have no external motivation for further improvement or because they are falling so far short that they despair of ever being able to reach the target.^7^ This selective response across organisations is suboptimal, as policy interventions achieve more when they shift the whole distribution rather than when they exert an influence over only a part of the distribution.^8^ The second way an organisation may respond to an incentive relates to the reporting of data, rather than the underlying data generating process. First, an organisation may be incentivised to take steps to collect and report data with greater diligence. Second, organisations may manipulate or distort data. There is a large literature on this unintended effect of incentives, which is referred to as ‘gaming’ in the economics literature.^1 9 10^ Data distortion results in unintended dispersion of incentives and does not benefit staff or patient well-being.

Better awareness of threshold effects could enable policymakers to apply different initiatives to motivate continued quality improvement across all organisations and to investigate potential instances of data distortion. The purpose of this paper is to illustrate a simple and inexpensive method to highlight where threshold effects occur. To illustrate this method, we examine a particular incentive in the CQUIN programme. The CQUIN programme commenced in 2009/2010 to improve care quality across NHS organisations in England.^1^ It contains time-specified performance targets across priority schemes, for example, to increase timely identification and treatment of sepsis and to increase uptake of the influenza vaccination. Where organisations fail to meet the targets, a portion of their contracted payment is held back, which sharpens the incentive through the mechanism of ‘loss aversion’.^11^

Here we focus on a CQUIN scheme designed to increase uptake of the seasonal influenza vaccination among frontline hospital staff. Monthly data on uptake across NHS hospital trusts are available from 2012 to 2013 onwards. Before the CQUIN scheme for influenza vaccination was initiated, the chief medical officer recommended a national indicative target of 70%, and local service commissioners were free (but not mandated) to set their targets and incentives.^12^ For example, before 2016, NHS England Durham Darlington and Tees Area Team and Barnsley Clinical Commissioning Group offered support to vaccinate all frontline staff to reach a particular target, but neither offered a financial incentive to do so.^13 14^ From 2016 to 2017 onwards, national CQUIN targets and incentives were set.^15^ The targets set to release full payments fluctuated over the four vaccination seasons covered by the national CQUIN, starting at 75%, dropping to 70% before reverting to 75% and then climbing further to 80%.^16^ Additional lower targets were set to release partial payments, starting at a threshold vaccination rate of 50%.^17^ While there is no official explanation for the threshold level, or reasons for a change in the threshold, it seems likely that the intention was to set the performance level for payment so as to motivate trusts to take positive action to meet this target, without setting it so high as to induce a sense of futility. The variations in the target for full payment were likely influenced by previous performance levels. In terms of the amount of money involved, the CQUIN payment for performance relating to the influenza vaccination programme was set at 0.25% of healthcare income for trusts. Based on consolidated accounts from NHS Provider account held by NHS England for 2018/2019,^18^ the total healthcare income for NHS trusts was £84.7 billion. This equates to £565 million across 150 NHS providers, and so the average CQUIN payment for the staff influenza vaccination programme would be around £1.4 million. However, with variations in organisational size, this would typically range between approximately £1 and £2 million per provider organisation.

The data on influenza vaccination uptake enable us to make four types of observation regarding threshold effects: the effect of the introduction of the national performance-based financial incentive system for full payment; the effect of subsequent changes in the annual target for full payment; the effect as the target date approaches; and the effects of partial payments at lower targets.

## Methods

### Study design

This is a retrospective observational study involving all NHS hospital trusts in England. We report our results in line with Strengthening the Reporting of Observational Studies in Epidemiology standards.^19^

### Data collection

Routine publicly available data were retrieved from Public Health England for acute, non-specialist NHS hospital trusts as defined by NHS Digital.^20^ We made use of data from 2012/2013 to 2019/2020 to cover similar 4-year periods before and after introduction of the national incentive. Data are presented as the total percentage staff uptake for the influenza vaccination season, which runs from September to February each year. The denominator is the number of staff defined as frontline healthcare workers, and the numerator is the number of these staff who were vaccinated.^21^ The data are submitted monthly during each vaccination season as cumulative totals. The number of trusts included varied per year, from 127 to 137, due to organisational changes, such as mergers and creations of new organisations.

### Analysis of threshold effects

For each vaccination season and for each full or partial payment target, we plotted the distribution of the vaccination performance to target ratios reported by hospital trusts. McCrary’s density test^6 22 23^ was applied to these distributions to determine whether there is discontinuity in the density function around the target set in each case. McCrary’s density test is a non-parametric method used in regression discontinuity analysis to identify whether there may have been manipulation in a continuous variable. It is a two-stage process; the first being to generate a histogram of the data, and the second involves fitting a local linear regression to the histogram on each side of the threshold separately, effectively smoothing the histogram. The counts within the histogram bin are the outcome variable for the regression model, and the midpoint value of the bin is the explanatory variable. McCrary also recommended an algorithm to determine the most appropriate bin size for the histogram, as these need to be carefully chosen to ensure that no bin covers the value where discontinuity may occur. A Wald test is then conducted with the null hypothesis that the discontinuity is zero.

The analyses were conducted using DCdensity in Stata V.15.1.^23^ Results are reported in terms of the ‘log difference in height’, which is the difference between the logarithms of the estimated density values on either side of the target. This serves as a discontinuity estimate. We also report the SE of the log difference in height estimate. The corresponding p value for the hypothesis test described previously is also reported in each case, and the Benjamini-Hochberg procedure was applied with a false discovery rate of 0.05. Plots for exploratory analyses were produced using ggplot2 in R.^24^

The above statistical method was applied to each of the three study objectives, which are to examine:

1. The effect of the introduction of the national performance-based financial incentive system.
2. The effect of subsequent changes in the targets for full payments; and
3. The effect of lower targets performance levels for partial payments.

To investigate the monthly results in more detail, we tested the hypothesis that trusts are targeting the number of vaccinations they need to perform in order to meet the full payment threshold. In this case, the running variable was defined as:

$$D_{t}=\frac{y_{t+1}-y_{t}}{T-y_{t}}$$

where $y_{t}$ is the cumulative proportion of vaccinations to month *t*, and *T* is the vaccination target level. This statistic has a value above 1 if an organisation has done what is required to meet the target during month *t* and a value between 0 and 1 if a trust is still falling short of the target by the start of month *t*+1. Hence, when looking at the distribution of $D_{t}$ values across trusts McCrary’s test will detect a discontinuity around one if trusts are doing just what is required to meet the target in that month.

### Patient involvement

No patients were involved in setting the research question or the outcome measures, nor were they involved in design or implementation of the study.

## Results

Table 1 summarises the percentage of trusts achieving the payments pre-CQUIN and post-CQUIN from 2012/2013 to 2019/2020, along with the number of NHS hospital trusts included each vaccination season. The targets set for full and partial payments are indicated. During the period of this study, the minimum threshold for full payment was 70%, which was raised to 80% by 2019/2020. Note that several targets are set for different partial payments, ranging from 50% to 65%.

**Table 1:** Percentage of trusts achieving targets pre-CQUIN and post-CQUIN

	Year	No. of Trusts*	Target(s) for partial payment (% of payment)	% of trusts meeting target(s) for partial payment	Target for full payment	% of trusts meeting target for full payment
Pre- CQUIN	2012/2013	135	–	–	70%†	9
	2013/2014	134	–	–	70%†	31
	2014/2015	137	–	–	70%†	24
	2015/2016	132	–	–	70%†	14
Post- CQUIN	2016/2017	134	65% (50)	64	75%	43
	2017/2018	129	50% (25)	99	70%	74
			60% (50)	91		
			65% (75)	83		
	2018/2019	127	50% (25)	99	75%	60
			60% (50)	91		
			65% (75)	83		
	2019/2020	134	60%–80% (graduated)	95‡	80%	56

Table created by the authors.

*During the study period, new NHS organisations were created and others merged. These changes are tracked by NHS England and NHS Digital with amendments being made to the national datasets accordingly. This accounts for variations in the number of trusts in the table.

†Indicates the recommended level of the locally agreed targets. All other targets are national CQUIN targets.

‡95% of trusts reached 60% vaccination uptake, which was the lower end of the graduated scale for partial payment.

CQUIN, Commissioning for Quality and Innovation.

While the vaccination season starts in September and ends in February, most vaccinations are delivered by the end of November (see online supplemental figure 1). This pattern remains consistent across all vaccination seasons. For example, half of the trusts had administered 84% or more of their 2019/2020 end-of-season total vaccinations before the end of November 2019.

10.1136/bmjqs-2021-013671.supp1 Supplementary data

### Effect of the introduction of the national performance-based financial incentive system

**Figure 1:**
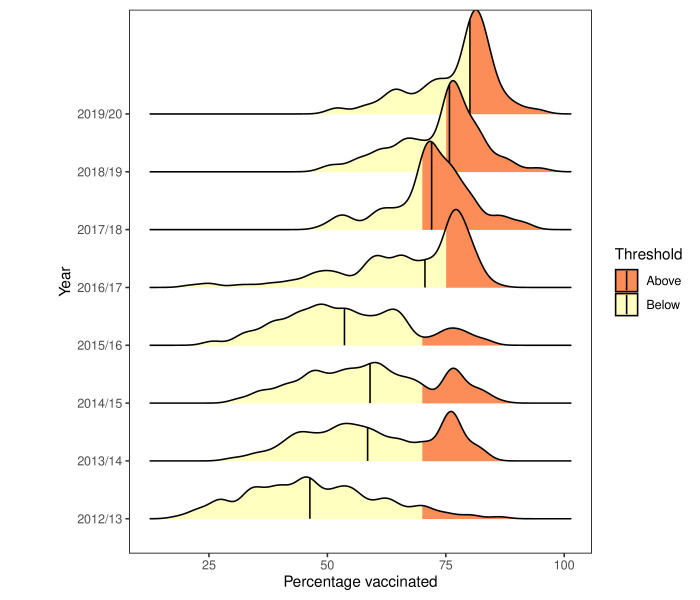
Ridgeline plot showing distributions of final influenza vaccination percentages for acute hospital trusts for winter seasons from 2012/2013 to 2019/2020. Each distribution is coloured according to whether the achieved percentages are above (orange) or below (yellow) the top threshold for that year. The median of each yearly distribution is indicated with a vertical black line. Created by the authors.

The density plots in figure 1 show the annual distributions of vaccination uptake reported by hospital trusts by the end of the vaccination seasons. In the 4 years before the national CQUIN incentive was set (2012/2013 to 2015/2016), the distributions are rather flat. The median performance levels vary from 46% to 58%, never exceeding the 70% suggested by the chief medical officer. In the 4 years after the national CQUIN was set, the distributions are more peaked. The median performance levels are higher than previously (71%–80%) and exceed the incentivised targets in three of the four seasons.

**Figure 2:**
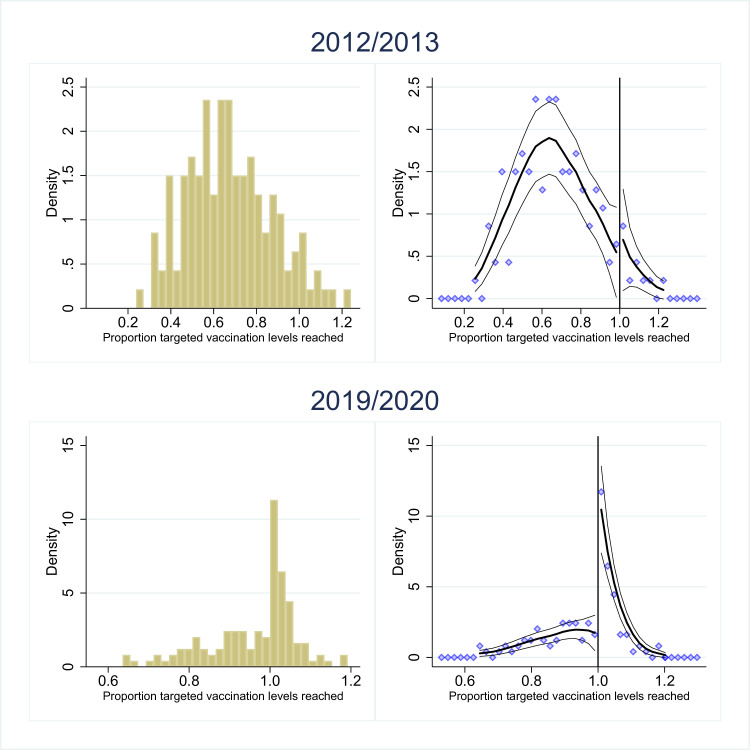
Histogram for end-of-season vaccination percentages reported by acute hospital trusts (left hand column) along with the predicted values and their 95% CIs from the local linear regressions fitted to the same binned data as part of McCrary’s density discontinuity test (right-hand column). Years shown are 2012/2013 (in the top row) and 2019/2020 (in the bottom row). Data have been transformed so that the x-axis shows vaccination percentage divided by the full payment threshold value for each influenza vaccination season, and therefore, the threshold for the purposes of McCrary’s test is at 1.0. Created by the authors.

The results of McCrary’s test are illustrated for the initial pre-CQUIN season (2012/2013) and the final post-CQUIN season (2019/2020) in figure 2. The left-hand panel contains histograms of the percentage/threshold ratios for vaccinations across all trusts and the right-hand panel contains local linear regressions fitted to the density of trusts in the bins on either side of one. The histogram for the pre-CQUIN season is flatter, with no obvious (or significant) discontinuity at the 70% target. In contrast, in the post-CQUIN season, the highest density of hospital trusts lies just to the right of one showing that a large number of trusts narrowly met the CQUIN target of 80% for full payment in 2019/2020. Similar plots for all seasons are presented in online supplemental figures 2–7. Note that the same patterns appear, such that threshold effects do not emerge in any of the pre-CQUIN seasons and does emerge in all the post-CQUIN seasons.

### Effect of subsequent changes in the targets for full payments

**Figure 3:**
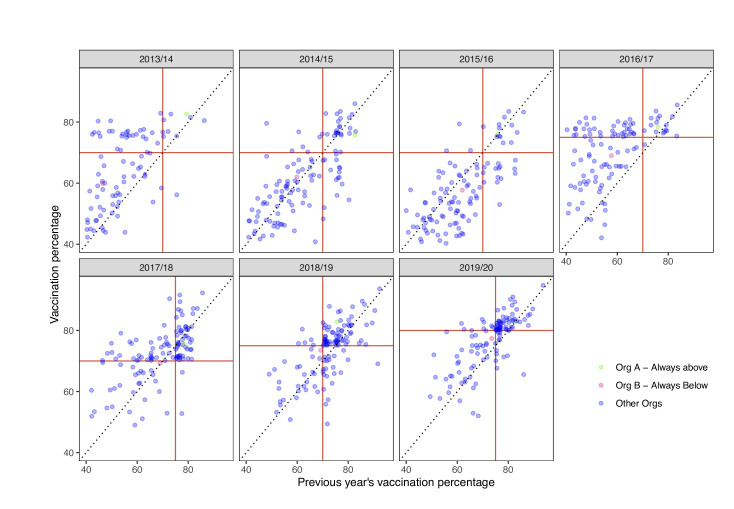
Comparison of vaccination percentages achieved by hospital trusts in consecutive years. The vaccination percentage in the year under consideration is plotted against the previous year’s vaccination percentage in each subplot. Red lines indicate the values of the threshold percentage for the two consecutive years (as detailed in table 1), so that points in the top-right quadrant formed by the red lines represent trusts achieving above the threshold in both years. The black dotted line indicates y=x. Of the 21 Trusts who achieved 0%–2% above the threshold in 2016/2017, nine (43%) also achieved 0%–2% above the threshold in 2017/2018. Of the 30 Trusts who achieved 0%–2% above the threshold in 2017/2018, 11 (36%) also achieved 0%–2% above the threshold in 2018/2019. Of the 35 Trusts who achieved 0%–2% above the threshold in 2018/2019, 18 (51%) also achieved 0%–2% above the threshold in 2019/2020. Created by the authors.

Threshold effects track the target level across all 4 years following introduction of the national CQUIN as it changes in the sequence 75%, 70%, 75% and 80% (figure 2 and online supplemental figures 5–7). The particular hospital trusts narrowly achieving the CQUIN targets (defined here as reaching 0–2 percentage points above the threshold) has remained markedly stable. Comparing the performance of hospital trusts across consecutive years shows that about 40% of trusts narrowly clearing the threshold in one vaccination season narrowly cleared it in the subsequent vaccination season. In figure 3, this is seen from the cluster of points just above and to the right of the intersection of the red lines in the plots for 2017/2018 onwards. Note that this cluster is seen both when the target had increased since the previous year (2018/2019 to 2019/2020 and 2017/18 to 2018/2019) and when it had decreased (2016/2017 to 2017/2018). Two trusts are tracked in figure 3 for illustrative purposes; one that was consistently below the threshold and one that was consistently above the threshold. In online supplemental figure 8, performances of trusts against the target are tracked over time.

One hundred and sixteen hospital trusts provided information every year on their vaccination rates since the introduction of the national CQUIN. Of these, 36 (30.2%) were consistently above the target threshold and 22 (18.5%) were consistently below the target threshold; the remaining 51.2% were above in some years and below the thresholds in others. There were no significant differences in these groups in the number of patients seen, admissions, full-time equivalent staff, proportion of completed episodes with no procedure, the proportion of patients seen who were from an urban area, nor the number of sites each organisation owns (online supplemental table 1).

### Changes in threshold effect month by month as the target date approaches

Performance reported in table 1 and figures 1 and 2 relates to end-of-season vaccination uptake, as payment is based on end-of-season numbers. However, as data are available for each month during the influenza season, we were also able to look for evidence of threshold effects in the cumulative data for each month. The results of McCrary’s test for discontinuity in the density of hospital trusts at the target are summarised in table 2. There was no evidence of threshold effects in any of the months for the years before the national CQUIN was introduced (p>0.1 in all cases). As previously noted, there was strong evidence of threshold effects in end-of-season performance in all four post-CQUIN years (p<0.001 in all cases). With the exception of the first year post-CQUIN (2016/2017), there was weak or no evidence of threshold effects on or before the end of December in each season.

**Table 2:** Log difference in height and p value from McCrary’s test for discontinuity at the full payment threshold for winter season influenza vaccination uptake at acute trusts

Year	October		November		December		January		February	
	Log difference in height (SE)	P value	Log difference in height (SE)	P value	Log difference in height (SE)	P value	Log difference in height (SE)	P value	Log difference in height (SE)	P value
2012/2013	△	△	0.0 (0.9)	0.677	0.6 (0.9)	0.489	–	–	–	–
2013/2014	△	△	−0.8 (0.8)	0.290	0.4 (0.6)	0.432	−0.0 (0.9)	0.978	–	–
2014/2015	△	△	0.3 (0.7)	0.700	−3.1 (2.1)	0.148	0.9 (0.8)	0.287	0.1 (0.9)	0.933
2015/2016	△	△	0.0 (1.2)	0.997	0.5 (0.8)	0.583	−0.1 (0.8)	0.853	0.2 (0.9)	0.776
2016/2017	△	△	1.5 (0.7)	0.193	3.2 (1.0)	**0.001**	2.8 (0.8)	**<0.001**	2.4 (0.6)	**<0.001**
2017/2018	0.9 (1.2)	0.448	0.1 (0.4)	0.795	0.7 (0.4)	0.066	1.4 (0.5)	**0.005**	2.1 (0.6)	**<0.001**
2018/2019	1.4 (1.1)	0.308	1.5 (0.7)	0.248	0.7 (0.4)	0.114	1.2 (0.5)	**0.007**	2.3 (0.6)	**<0.001**
2019/2020	△	△	△	△	−0.1 (0.5)	0.826	0.8 (0.4)	0.247	2.0 (0.5)	**<0.001**

△=too few observations on one side of the cut-off to perform the test.

–=no data available for that month.

P values highlighted in bold are significant after Benjamini-Hochberg adjustment with a false discovery rate upper bound of 5%.

Table created by the authors.

We also looked from a second perspective at how trusts were reacting to the target in different months, hypothesising that there would be a relationship between the percentage of staff vaccinated in a month and how far a trust was from the full payment threshold at the start of that month. We tested this by calculating the percentage of staff vaccinated in a month as a proportion of the distance from the target in percentage points at the end of the previous month for each trust. The estimated distribution of these values is shown in online supplemental figure 9 for each pair of consecutive months in each vaccination season. Between October and November of each season, the peak appears to be between zero and one, indicating that many trusts are moving towards but not reaching the target. Between January and February, there is consistently a peak at zero (indicative of no or very few vaccinations taking place), followed by a second peak above one (indicative of trusts vaccinating just enough staff to achieve the target). Hence, we see evidence of trusts targeting the number of vaccinations they need to perform in order to meet the full payment threshold. This is consistent with the hypothesis that trusts will react as the target date approaches.

### Effects of lower target performance levels for partial payments

A single partial payment target was introduced with the full payment target in 2016/2017. Multiple partial payment targets were then introduced in 2017/2018 and maintained for 2018/2019 (table 1). We conducted McCrary’s density test at each of these partial payment targets. In 2017/2018 and 2018/2019, 99% of hospital trusts reached the target value of 50% vaccination uptake required for achieving 25% of the payment; therefore, there were not enough data points below the target to conduct the test. For the remaining partial targets, McCrary’s test suggested no evidence of threshold effects (p>0.1 in each case; online supplemental table 2).

## Discussion

### Headline findings

Uptake of vaccination more than doubled after the CQUIN was introduced from 2016/2017 onwards. Hospitals across England reacted to the CQUIN target, either by reporting what they were already doing or by improving vaccination rates. Density tests of recorded uptake showed statistically significant evidence of threshold effects in NHS hospital trusts in all 4 years examined since the national CQUIN payment targets came into effect (2016/2017 onwards). These threshold effects were absent in the preceding years prior to the target. It is argued that threshold effects (along with instrumental variables) are ‘next best’ to randomisation in determining cause–effect relationships. The finding that not only did the threshold effect appear in all 4 years but that it tracked the moving threshold level and appears only in the later months of the observation period makes a compelling case that the threshold incentive caused threshold effect to appear. The alternative explanation is that the observed data are the result of reporting behaviour not of any underlying change in the data generation process, as we now discuss.

### Could the observed pattern be an artefact of data submission rather than an underlying process?

The observed pattern was of an *increase* in recorded vaccinations following introduction of the national incentive. In order for reporting to be the reason for ‘bunching’ it would be necessary for there to have been a simultaneous decrease in reporting among hospitals that had cleared the threshold. We think that to produce such a pattern in the face of no change in the underlying vaccination rate is implausible. Furthermore, it is a legal requirement to couple vaccination to data recording for vaccine stewardship purposes. For both the stated reasons, we do not think the ‘bunching’ or threshold effect can be convincingly ascribed to withholding data that had been collected. This leaves open the possibility that data were manipulated, say by including in the numerator people who did not appear in the denominator—a point to which we will return. Alternatively, the hospitals ceased further efforts to elicit vaccinations once they had cleared the threshold.

### Our study in relation to other literature

We have conducted a scoping review and collected references suggested by experts, including a helpful journal reviewer. There is a vast literature on incentives and pay-for-performance schemes, including two recent reviews.^25 26^ There were many articles on the incentivising and disincentivising effects of different threshold levels (eg, Dowd *et al* 27; Kontopantelis *et al* 28) and on removing thresholds (eg, Wang *et al*).^29^ There are also numerous scholarly articles describing how the design of incentives affects their performance, most notably Doran *et al* and Oxholm *et al* 30 31 and on gaming. We found only a small number of articles dealing specifically with the threshold effect itself. Gruber *et al* 32 found that bunching occurs just before the 4-hour accident and emergency department waiting time target in England but do not use threshold analysis per se. Takaku *et al* also identify bunching of recorded admissions just before midnight, especially in private hospitals, presumably to obtain an extra day of inpatient payment.^33^ We are also aware of another study in the public health field.^6^ Our study seems novel insofar as it identifies the threshold effect and studies both how the phenomenon tracks a moving threshold level and the relationship of the threshold effect to the approaching target date.

### What causes the threshold effect?

The ‘bunching’ immediately after the threshold level could be caused by deliberate data manipulation or ‘gaming’ (we shall call this the ‘manipulation hypothesis’). Alternatively, or in addition, it could be caused by altered behaviour, whereby no further effort is made once the threshold has been cleared (we shall call this the ‘effort hypothesis’). While the routine data used in this study cannot distinguish these two possible causes, the fact that one or other or both must exist is of theoretical and policy importance.

### Implications of the effort hypothesis

The effort effect has epidemiological and behavioural implications.

From an epidemiological point of view, interventions with threshold effects are suboptimal, since more can be achieved by improving a distribution as a whole than by selective action around a threshold.^8^ The policy response to this could be to redesign the incentive to include multiple thresholds or a continuous scale. From a behavioural point of view, the effort hypothesis could be taken as a signal that extrinsic motivation is crowding out intrinsic motivations. This could be accepted as an unavoidable unintended effect of an effective intervention. However, it provokes a question about other potential interventions. These policy implications are discussed in further detail below.

### Implications of the manipulation hypothesis

Manipulation of data to reach a target, if that is at least in part responsible for the threshold effect, is a concern. Not only does it vitiate the purpose of the incentive and create a false sense of benefit, but a policy that rewards bad behaviour is not acceptable. The possibility of manipulation cannot be ignored for two reasons: there is ample scope for manipulation of data to occur and the literature is replete with examples thereof.

Regarding opportunity, the instructions provided to NHS organisations on how they should collect vaccination data allows latitude. Choices can be made regarding who is considered frontline staff; some staff types (such as nurses in training) may be included in the numerator but not the denominator, for example. Some hospitals with limited electronic capacity sample only a portion of their staff.^21^

Regarding the literature on the subject, ‘gaming’ is common when there is a financial incentive. Bevan and Hood conclude that performance-based incentive systems in the NHS encourage ‘gaming’, and they provide examples across NHS services.^34 35^ They propose several mechanisms that may mitigate gaming: (1) do not specify how performance must be measured far in advance as such specification almost invites gaming early on—an idea further supported by Oxholm *et al* 31; (2) when gaming is evidenced, then action must be taken to ensure gaming does not become an organisational norm; and (3) increase face-to-face interactions/transparency between providers and the public. In a similar vein, Propper *et al* evidence gaming in NHS services around waiting times.^36^ However, the extent to which these problems can be mitigated remains uncertain.^31^

### Partial payment thresholds at lower levels of performance

Organisations may fail to improve when they are falling well below the trajectory to reach the target level, and they feel that there is too much ground to be made up. This was likely the motive for the partial payment targets described here. The idea is that those at lower levels will not be disincentivised by a sense of futility. However, we found no evidence that the partial incentive had any effect. We speculate that this might have been for three (non-exclusive) reasons. First, the ‘dose’ of the partial incentive might have been too low to elicit a response. Second, organisations at low levels might have had weaker management systems such that they were less able to rise to the challenge. Third, some organisations might not have been aware of the partial payment option.

### Policy implications

The policy options are to: (1) tolerate the problems with the incentive system because it seems to be working; (2) improve the design of the system; or (3) use an alternative intervention to drive up rates.

The first option may be seen as optimal since the intervention did increase vaccine uptake and, as with a clinical intervention, the disadvantages might represent a good trade-off. To put this another way, the threshold effect might be a small price to pay for the advantages of the incentive system. Such an opinion might be more justified if gaming could be excluded.

The second option, improving the design of the incentive, seems to have been exhausted since both multiple thresholds and a continuous scale have been used. Increasing the quantum (or dose) of the incentive might work, but there is a limit to how much society is willing to pay for a unit of improvement. Other methods introduce complexity, which is inimical to successful incentives.

The third option, alternative interventions, begs the question of what these may be. Brewer *et al*’s^37^ scoping review describes three types of interventions to increase uptake where vaccines are available and affordable. The first targets thoughts and feelings. Evidence supporting these interventions are inconsistent and backfire effects are common.^38^ The second type of intervention targets social norms. Evidence supporting the effects of social norms largely comes from retrospective or descriptive studies, where people who live together seem to share similar attitudes towards vaccination.^39 40^ Experimental evidence supporting the effectiveness of social norms interventions is inconsistent. A simple letter-based social norms intervention had no effects on hospital staff uptake of influenza vaccinations (uptake was 43% in all groups),^41^ but a multifaceted social norms intervention increased uptake by 12 percentage points (from 20% to 32%). The third type of intervention targets behaviour directly. Here the aim is to support provaccination intentions, reduce barriers or shape behaviour. The current CQUIN intervention fits this category, as it aims to shape behaviour through financial incentives, although at the organisational level. Evidence supporting the effectiveness of direct behavioural interventions is favourable. For example, providing people with a prescheduled ﬂu shot appointment time has been shown to increase influenza vaccinations for patients by 11 percentage points (from 5% to 16%)^42^ and for hospital staff by 12 percentage points (from 16% to 28%). Behavioural interventions may require vaccinations to access other opportunities; for example, many hospitals in the USA mandate staff to receive the influenza vaccine. A systematic review of such mandates finds large increases from preintervention to postintervention (from a mean of 72%–94%).^43^ Mandates require fair enforcement and are coercive.^44 45^ There seems to be a genuine trade-off between positive liberty for employees and negative liberty for patients and other staff members that they may affect. In times of national emergencies, such as a COVID-19 or influenza pandemic, coercion might be more justified than in times where no emergency is anticipated.

### Limitations and next steps

One limitation of this analysis is that it is restricted to one type of service provider: NHS hospital trusts. We do not examine information regarding community-based institutions, primary care, ambulance services and so on. Larger organisations are likely to have the managerial capacity to detect and react to an accumulating performance level that promises to fall short of the target. Likewise, institutions with sophisticated information systems can easily track performance on a rolling basis and may therefore be more reactive than those that rely on paper-based systems. In addition, qualitative interviews might reveal sensitive information about how data linked to financial incentives are managed and to understand human behaviour in response to thresholds that trigger financial rewards and penalties. It is also worth mentioning that there are a range of factors affecting compliance with processes such as influenza vaccination for staff. Such factors include the perceived severity of the identified potential influenza strain in a particular year. COVID-19 is another such factor that may be a ‘game-changer’ in affecting attitudes towards vaccination. In this respect, the possibility that the bunching of vaccination represents displacement of endogenous motivation is a concern, as is evidence that withdrawing an incentive can lead to a sharp deterioration in performance.^46^ Whether incentive systems will be used with respect to COVID-19 and how effective they will prove remains to be seen, but there is a hint in our data that relying on external motivation may not be the full answer. Once compliance reaches its theoretical maximum, then the concept of a threshold effect is vitiated.

In this study, we have only analysed data for frontline staff influenza vaccinations. There are many other performance indicators, which could be studied to analyse the behaviour of trusts when faced with financial incentives.

## Conclusion

Our study was conducted with nationally reported data and the clear presence of threshold effects show that hospitals take note of the incentive and take action to meet the target. As stated in the introduction, the presence of threshold effects is silent on what caused it. Nevertheless, we could find clues to causation in the data for at least a proportion of hospitals. The presence of threshold effects gives policy makers some indication of how the system is responding beyond the headline figure of compliance. This in turn may lead to some enquiry into what may be learnt and how the implementation of the incentive could be improved.

## Data Availability

This study used publically available data which can be downloaded from https://www.gov.uk/government/collections/vaccine-uptake.
