# Supplementary material for: Examining organisational responses to performance-based financial incentive systems: a case study using NHS staff influenza vaccination rates from 2012/2013 to 2019/2020
Source: BMJ Qual Saf. 2021 Sep 28;31(9):642–51. doi: 10.1136/bmjqs-2021-013671 (PMC9411890; doi:10.1136/bmjqs-2021-013671)
Supplement: Supplementary data [file bmjqs-2021-013671supp001.pdf]

Examining organisational responses to performance-based financial incentive systems: A case study using NHS staff influenza vaccination rates from 2012/13 to 2019/20

SUPPLEMENTARY FIGURES AND TABLES

Supplementary Figure 1: Boxplots showing distribution of percentage of frontline staff vaccinated by month for acute hospital trusts. Note that the October figure includes vaccinations administered in both September and October.

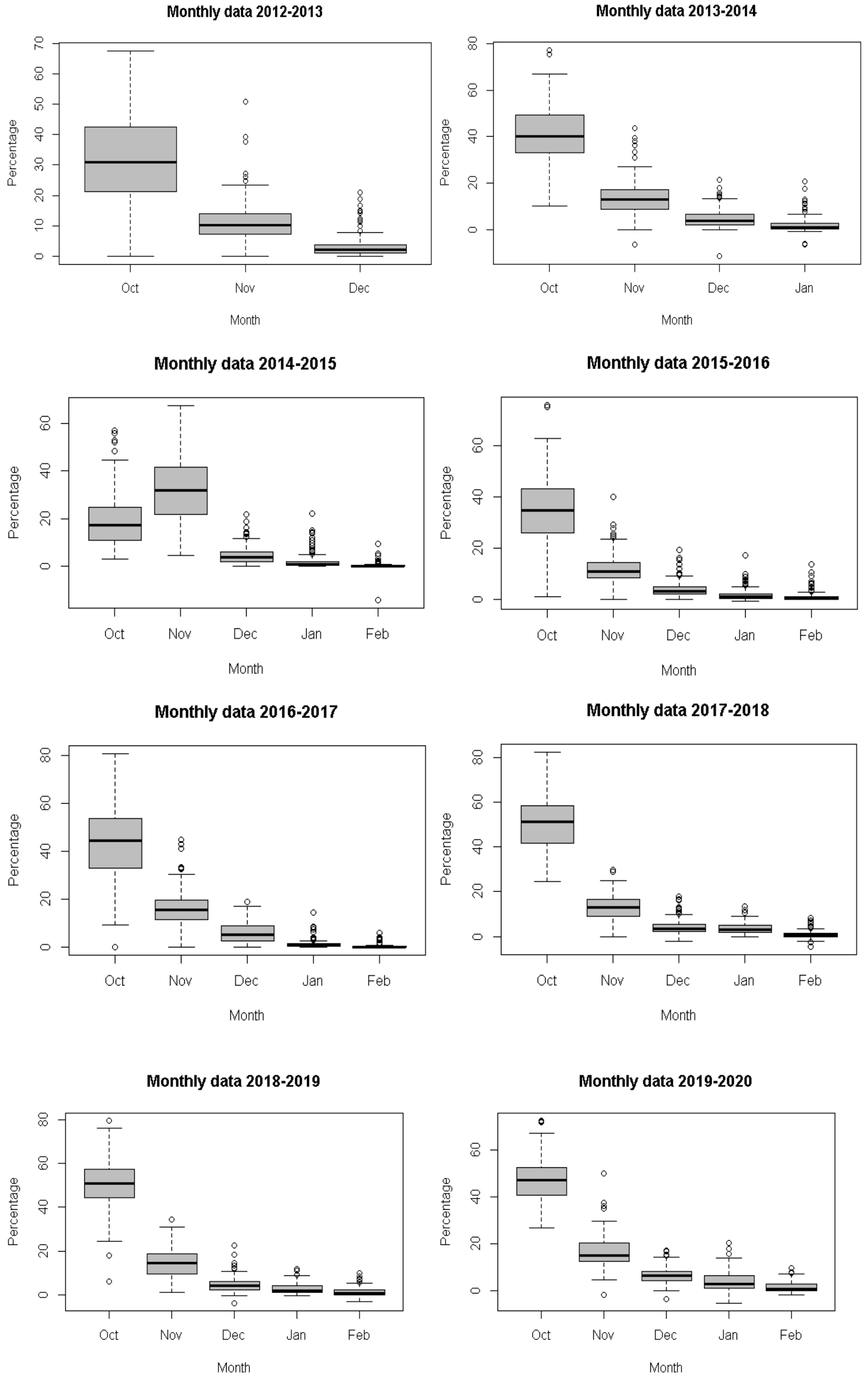

Examining organisational responses to performance-based financial incentive systems: A case study using NHS staff influenza vaccination rates from 2012/13 to 2019/20

Supplementary Figure 2: Histogram showing density of hospital trusts against end-of-season vaccination performance in 2013/14 along with the predicted values and their 95% confidence intervals from the local linear regressions fitted to the same binned data as part of McCrary's density discontinuity test (right hand column). Data have been transformed so that the x axis shows vaccination percentage divided by 70% (the recommended threshold value), and therefore the threshold for the purposes of McCrary's test is at 1.0.

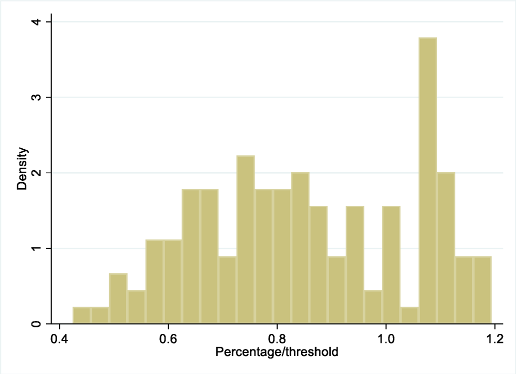

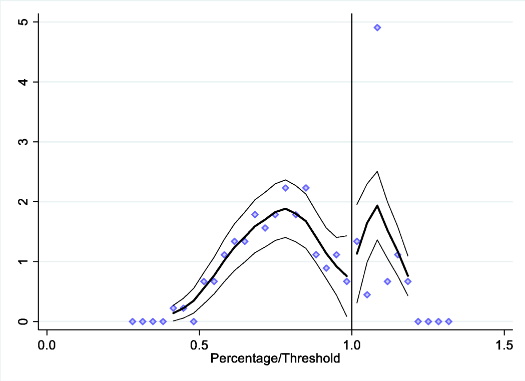

Examining organisational responses to performance-based financial incentive systems: A case study using NHS staff influenza vaccination rates from 2012/13 to 2019/20

Supplementary Figure 3: Histogram showing density of hospital trusts against end-of-season vaccination performance in 2014/15 along with the predicted values and their 95% confidence intervals from the local linear regressions fitted to the same binned data as part of McCrary's density discontinuity test (right hand column). Data have been transformed so that the x axis shows vaccination percentage divided by 70% (the recommended threshold value), and therefore the threshold for the purposes of McCrary's test is at 1.0.

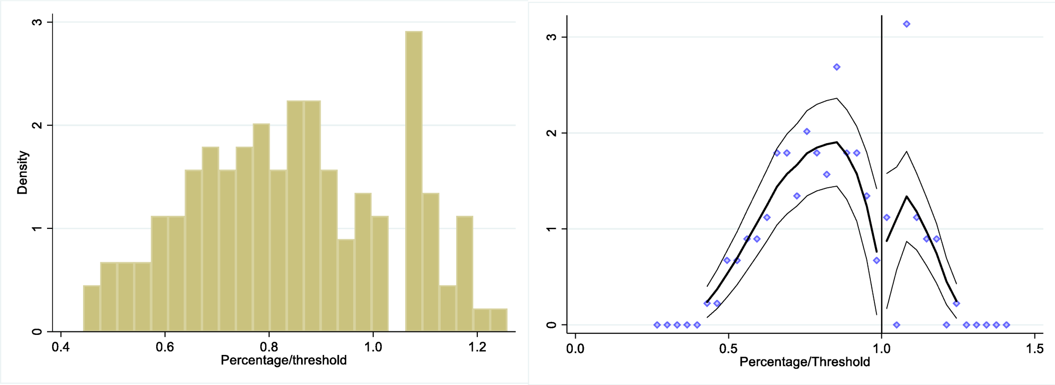

Examining organisational responses to performance-based financial incentive systems: A case study using NHS staff influenza vaccination rates from 2012/13 to 2019/20

Supplementary Figure 4: Histogram showing density of hospital trusts against end-of-season vaccination performance in 2015/16 along with the predicted values and their 95% confidence intervals from the local linear regressions fitted to the same binned data as part of McCrary's density discontinuity test (right hand column). Data have been transformed so that the x axis shows vaccination percentage divided by 70% (the recommended threshold value), and therefore the threshold for the purposes of McCrary's test is at 1.0.

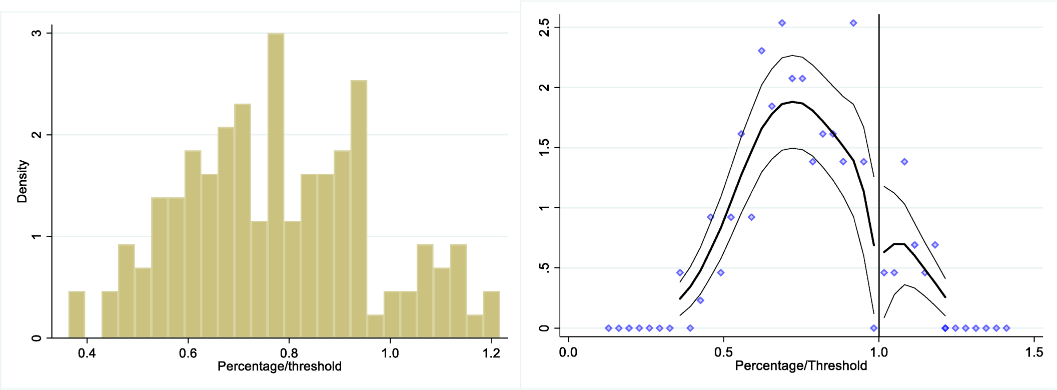

Examining organisational responses to performance-based financial incentive systems: A case study using NHS staff influenza vaccination rates from 2012/13 to 2019/20

Supplementary Figure 5: Histogram showing density of hospital trusts against end-of-season vaccination performance in 2016/17 along with the predicted values and their 95% confidence intervals from the local linear regressions fitted to the same binned data as part of McCrary's density discontinuity test (right hand column). Data have been transformed so that the x axis shows vaccination percentage divided by 75% (the full payment threshold value), and therefore the threshold for the purposes of McCrary's test is at 1.0.

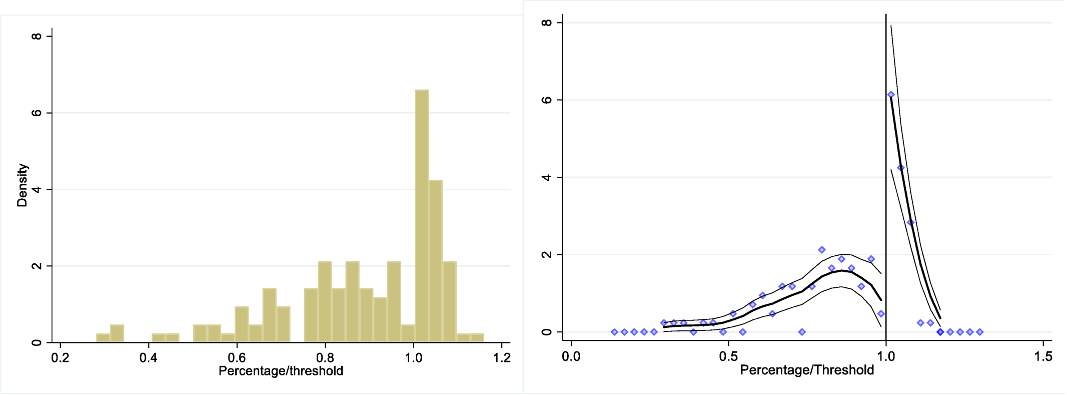

Examining organisational responses to performance-based financial incentive systems: A case study using NHS staff influenza vaccination rates from 2012/13 to 2019/20

Supplementary Figure 6: Histogram showing density of hospital trusts against end-of-season vaccination performance in 2017/18 along with the predicted values and their 95% confidence intervals from the local linear regressions fitted to the same binned data as part of McCrary's density discontinuity test (right hand column). Data have been transformed so that the x axis shows vaccination percentage divided by 70% (the full payment threshold value), and therefore the threshold for the purposes of McCrary's test is at 1.0.

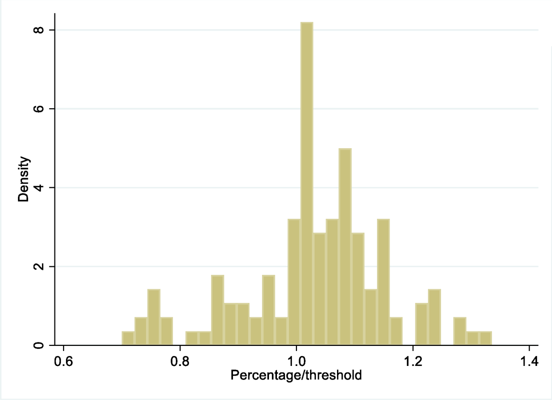

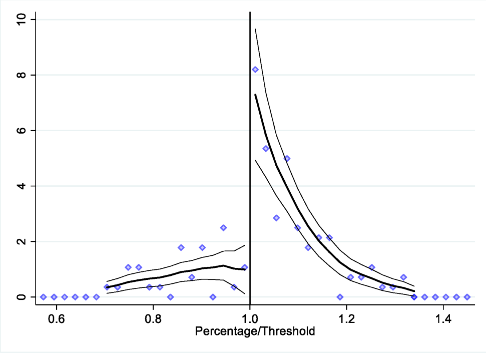

Examining organisational responses to performance-based financial incentive systems: A case study using NHS staff influenza vaccination rates from 2012/13 to 2019/20

Supplementary Figure 7: Histogram showing density of hospital trusts against end-of-season vaccination performance in 2018/19 along with the predicted values and their 95% confidence intervals from the local linear regressions fitted to the same binned data as part of McCrary's density discontinuity test (right hand column). Data have been transformed so that the x axis shows vaccination percentage divided by 70% (the full payment threshold value), and therefore the threshold for the purposes of McCrary's test is at 1.0.

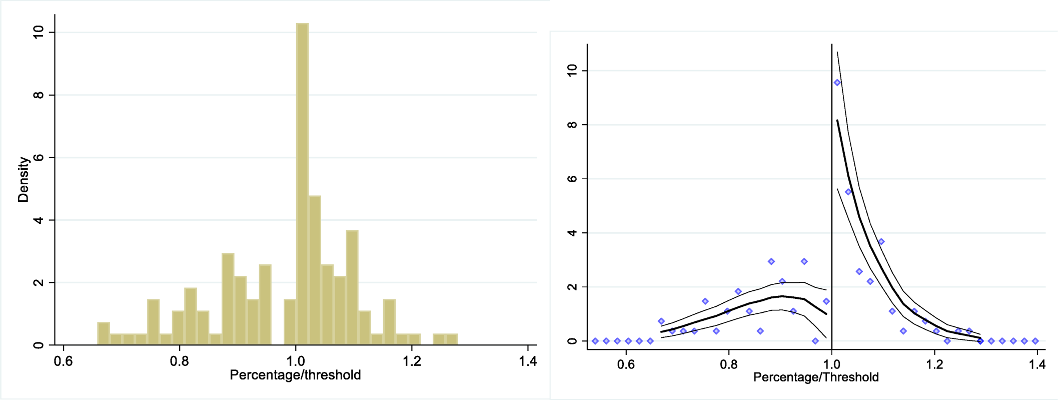

Examining organisational responses to performance-based financial incentive systems: A case study using NHS staff influenza vaccination rates from 2012/13 to 2019/20

Supplementary Figure 8:Tracking trusts' performance against the vaccination target over time.

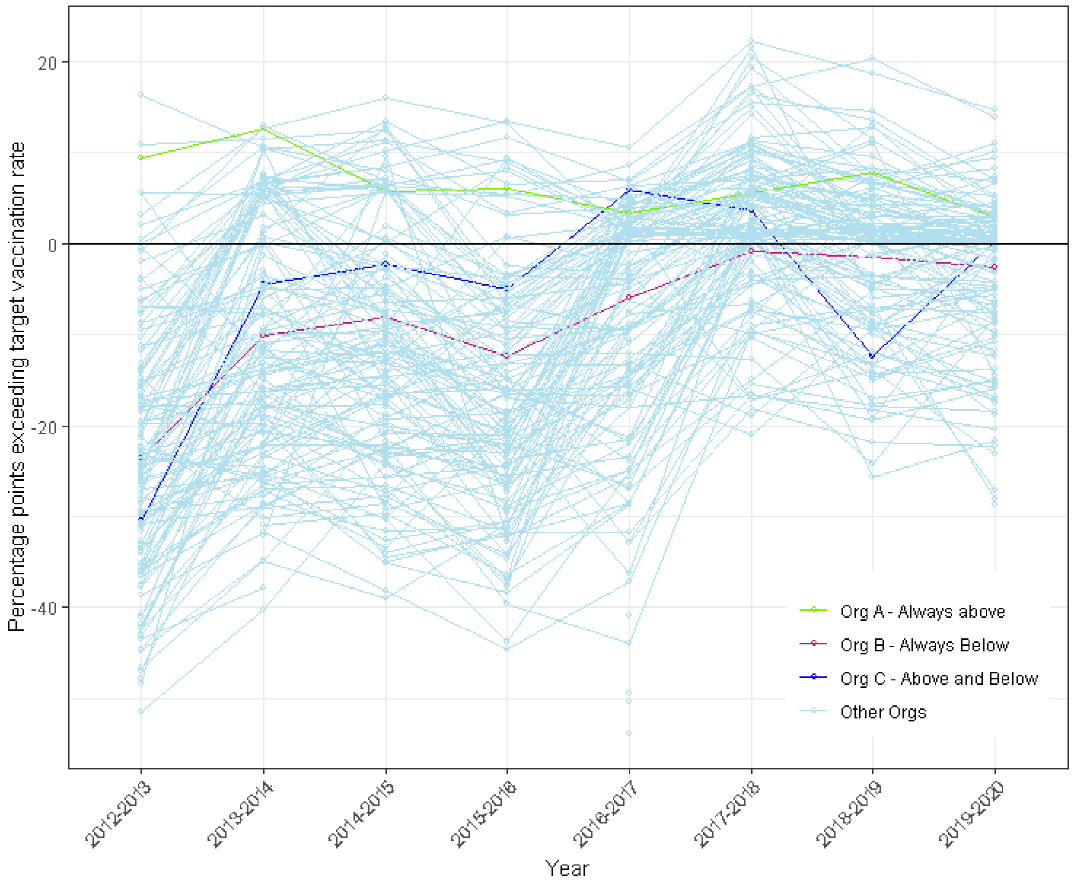

Examining organisational responses to performance-based financial incentive systems: A case study using NHS staff influenza vaccination rates from 2012/13 to 2019/20

Supplementary Figure 9: The x-axis is the percentage of staff vaccinated from one month to the next, divided by percentage of vaccinations needed to reach the threshold value at the end of the first month. Solid lines show predicted values and dotted lines show 95% confidence intervals from the local linear regressions fitted to the binned data as part of McCrary's density discontinuity test. Each colour corresponds to a pair of consecutive months during the vaccination season; x-axis has been truncated to between 0 and 5. A discontinuity around one is expected if organisations have vaccinated just enough staff in that month to meet the target threshold.

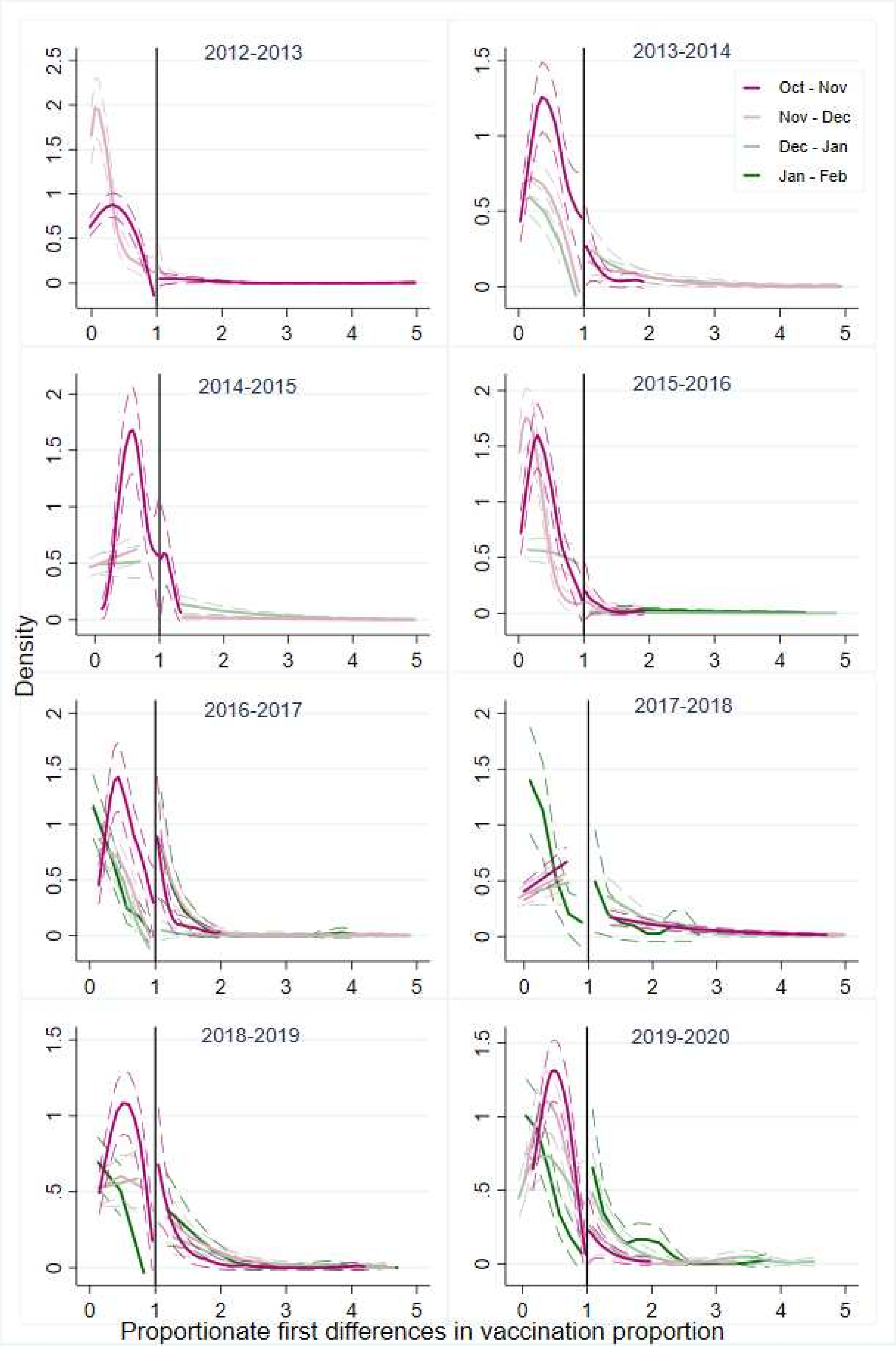

Examining organisational responses to performance-based financial incentive systems: A case study using NHS staff influenza vaccination rates from 2012/13 to 2019/20

Supplementary Table 1: Comparison of trusts who were consistently over the vaccine target for all fours years since the introduction of the CQUIN, with those who were consistently under in all four years or were over and under the target against measures from the NHS Digital Peer Finder Tool.

	Over all four years Median (IQR)	Mix over and under Median (IQR)	Under all four years Median (IQR)	p value
Patients seen	190550 (153170-297958)	213225 (164880-309725)	243708 (191365-301395)	p=0.4244
Admissions	898882.5 (668933-1241290)	892015 (676065-1387045)	1087830 (849405-1270815)	p=0.5087
Full time equivalent staff	5227.35 (3662.3-7125.3)	4510.1 (3431.6 - 6578.9)	5513.7 (4162.6-7681.7)	p=0.3060
Proportion of finished episodes with no procedure	0.44 (0.40-0.49)	0.43 (0.39-0.47)	0.42 (0.37-0.47)	p=0.2960
Proportion of patients seen who were from an urban area	0.82 (0.73-0.90)	0.82 (0.65-0.94)	0.78 (0.67-0.91)	p=0.7967
Number of sites	64 (38-124)	73 (39-121)	89.5 (61-141)	p=0.2680

Examining organisational responses to performance-based financial incentive systems: A case study using NHS staff influenza vaccination rates from 2012/13 to 2019/20

Supplementary Table 2: Results of McCrary's density discontinuity test at partial payment targets in 2016/17 – 2018/19

Year and partial payment targets (% Payment released)	Log difference in height (standard error*)	p value
**2016/17**		
65% (50)	0.3(0.5)	0.586
**2017/18**		
50% (25)	-	-
60% (50)	1.7 (1.1)	0.140
65% (75)	-0.0 (1.1)	0.994
**2018/19**		
50% (25)	-	-
60% (50)	1.2 (1.1)	0.272
65% (75)	4.8 (3.9)	0.211

Notes: Dashes (-) indicate too few observations on one side of the cut off to perform the test. Similarly, high values of the standard error indicate too few Trusts achieving vaccination percentages within the vicinity of the threshold to accurately estimate the log difference in height.
